# Supplementary material for: HybridoNet-Adapt: A domain-adapted framework for accurate lithium-ion battery RUL prediction
Source: PLoS One. 2025 Oct 31;20(10):e0335066. doi: 10.1371/journal.pone.0335066 (PMC12578170; doi:10.1371/journal.pone.0335066)
Supplement: Attachment — Submitted filename: Response_to_Reviewers.pdf [file pone.0335066.s001.pdf]

Response Letter

We sincerely thank Editor-in-Chief, Associate Editor, and the reviewers for their constructive, pertinent, and useful comments on our manuscript.

To Editor-in-Chief and Associate Editor

We are pleased to see insightful and constructive comments from the reviewers. In this resubmission, we have attempted to address the reviewers' concerns and revised the manuscript accordingly. Further details about all the changes we made can be found in our point-to-point response to the reviewers' comments below. In the revised manuscript, we marked the changes in yellow.

To Reviewer 1

On Page 3, the statement "Many existing methods predict RUL based on estimated maximum discharge capacity, representing the remaining life as a percentage of the nominal capacity" is actually the definition of SOH, not RUL. Please carefully distinguish between these two concepts. Accordingly, some references listed in Table 1 are not truly RUL prediction studies.

Response: Thank you for your comment. We have revised the statement to reflect the RUL concept. The updated text begins with "Historical data-independent methods estimate the future capacity trajectory ..." on Page 3 and "RUL can be assessed based on the number of remaining cycles" on Page 2. In addition, we have removed a reference that predicts cycle life rather than RUL from Table 1.

Comment 2: To further strengthen the introduction, it would be beneficial to include more recent and relevant works, such as DOI: 10.1016/j.jpowsour.2022.230975; 10.1016/j.est.2025.116024.

Response: Thank you for your comment. We have added the two references by updating Table 1 and including a paragraph beginning with "[31] proposed a transferable RUL prediction ..." in the Problem Statement and Potential section.

Comment 3: The manuscript currently lists five contributions. This number is excessive and somewhat redundant. It would be clearer and more impactful to condense and reorganize them into three key contributions.

Response: Thank you for your comment. We have updated the contributions and condensed them into three key points. The revised text begins with "We propose a historical data-independent RUL prediction framework for ...".

Comment 4: Section titles should not contain references or colons. Please revise accordingly.

Response: Thank you for your comment. We have remove the references and colons from two section titles (LSTM, Multihead Attention, NODE).

Comment 5: All equations should be numbered, and every variable used in the equations must be explained. Avoid using whole words as variable names in formulas.

Response: Thank for your comment. We have revised the equations in the Preliminaries section, ensuring that all equations are numbered and every variable is clearly defined, while avoiding the use of whole words as variable names.

Comment 6: In Figure 2, the arrows pointing from the MSE loss and MMD loss to the Input are confusing. Please check whether the statement is correct or explain the meaning of the arrows.

Response: Thank for your comment. We have revised Figure 2 by removing the arrows from the MSE and MMD loss blocks to the input, ensuring that the diagram accurately reflects the intended training flow.

Comment 7: It appears that target-domain labels may have been used from Page 7. If so, why not directly train and test using the target-domain data? Please clarify whether target labels are available, and if they are, discuss the meaningfulness of the domain adaptation task under this setting.

Response: Thank you for your comment. Yes, target-domain labels are available. To address this, we created HybridoNet (without domain adaptation), which consists of only a feature extractor and a target predictor and is trained solely on labeled target data. The updated text, beginning with "For validating the proposed domain adaptation used in HybridoNet-Adapt, we ...", has been added to the Proposed method / HybridoNet-Adapt: A Proposed RUL Prediction model with Novel Domain Adaptation section.

Comment 8: In many published works using the TRI dataset, models are typically trained on Batch 1 and tested on Batches 2 and 3. If target-domain labels were used to aid model training, then the task defined in this paper may be easier, and the results may not provide strong evidence that the proposed method is more effective than existing approaches.

Response: Thank you for your comment. We have added Figure 11(a), which compares our proposed HybridoNet-Adapt (146.52 RMSE) with the MSFEH 2025 state-of-the-art model (161.81 RMSE). This result shows that our proposed method, even when using target-domain labels, substantially outperforms the current state-of-the-art under this setting.

For the second dataset, we have removed the comparison with the Dual-input DNN because it

was conducted under a different experimental setup. Our proposed method follows the official split of the second dataset, with 55 LIBs for training and 22 LIBs for testing. In contrast, the Dual-input DNN work re-split the dataset, using 70 batteries for training and only 7 for testing, which makes its evaluation less generalizable.

Comment 9: Hyperparameters should be presented in a table for clarity and reproducibility.

Response: Thank you for your comment. We have included Table 2 for the hyperparameters, along with a paragraph explaining the notation, beginning with "The hyperparameters of the proposed HybridoNet-Adapt are summarized ...".

To Reviewer 2

Comment 1: The study lacks practical interpretability. Although the methodology is emphasized, the model predictions are not supported by physical explanations or visual analyses.

Response: Thank you for your comment. We acknowledge the importance of interpretability in bridging the gap between predictive performance and physical insights. We plan to integrate Physics-Informed Neural Networks in future work, as noted in the added text beginning with "In future work, we plan to explore Physics-Informed Neural Networks (PINNs) ..." in the Experiments and discussion Section.

Comment 2: The baselines are mostly traditional methods or relatively simple deep learning models, while more recent approaches such as advanced transfer learning or self-supervised learning are not systematically compared.

Response: Thank you for your comment. We have compared our model with recent state-of-the-art approaches. For the first dataset, we have added Figure 11(a), which compares our proposed HybridoNet-Adapt (146.52 RMSE) with the MSFEH 2025 state-of-the-art model (161.81 RMSE). This result shows that our proposed method substantially outperforms the current state-of-the-art under this setting.

For the second dataset, we have added the Structural Pruning 2024 state-of-the-art model in Figure 11(b). We also removed the comparison with the Dual-input DNN because it was conducted under a different experimental setup. Our proposed method follows the official split of the second dataset, with 55 LIBs for training and 22 LIBs for testing. In contrast, the Dual-input DNN re-split the dataset, using 70 batteries for training and only 7 for testing, which makes its evaluation less generalizable.

Comment 3: While different feature extraction modules and alignment losses are examined, the ablation study remains incomplete, as it does not explore alternative feature selection strategies or the sensitivity to different sampling window lengths.

Response: Thank you for your comment. We have added an ablation study (see Figure 5 and the accompanying text beginning with "Figure 5a presents the feature importance ranking derived

from XGBoost...") to compare our chosen statistical feature extraction method with alternative approaches.
